# Conotoxin Diversity in the Venom Gland Transcriptome of the Magician’s Cone, *Pionoconus magus*

**DOI:** 10.3390/md17100553

**Published:** 2019-09-27

**Authors:** José R. Pardos-Blas, Iker Irisarri, Samuel Abalde, Manuel J. Tenorio, Rafael Zardoya

**Affiliations:** 1 Departamento de Biodiversidad y Biología Evolutiva, Museo Nacional de Ciencias Naturales (MNCN-CSIC), José Gutiérrez Abascal, 2, 28006 Madrid, Spain; jpblas@mncn.csic.es (J.R.P.-B.); iker@mncn.csic.es (I.I.); saabalde@gmail.com (S.A.); 2 Departamento CMIM y Q. Inorgánica-INBIO, Facultad de Ciencias, Universidad de Cádiz, 11510 Puerto Real, Spain; manuel.tenorio@uca.es

**Keywords:** RNA-seq, D superfamily, A alpha 4/3, differential expression, O1 precursor phylogeny

## Abstract

The transcriptomes of the venom glands of two individuals of the magician’s cone, *Pionoconus magus*, from Okinawa (Japan) were sequenced, assembled, and annotated. In addition, RNA-seq raw reads available at the SRA database from one additional specimen of *P. magus* from the Philippines were also assembled and annotated. The total numbers of identified conotoxin precursors and hormones per specimen were 118, 112, and 93. The three individuals shared only five identical sequences whereas the two specimens from Okinawa had 30 sequences in common. The total number of distinct conotoxin precursors and hormones for *P. magus* was 275, and were assigned to 53 conotoxin precursor and hormone superfamilies, two of which were new based on their divergent signal region. The superfamilies that had the highest number of precursors were M (42), O1 (34), T (27), A (18), O2 (17), and F (13), accounting for 55% of the total diversity. The D superfamily, previously thought to be exclusive of vermivorous cones was found in *P. magus* and contained a highly divergent mature region. Similarly, the A superfamily alpha 4/3 was found in *P. magus* despite the fact that it was previously postulated to be almost exclusive of the genus *Rhombiconus*. Differential expression analyses of *P. magus* compared to *Chelyconus ermineus*, the only fish-hunting cone from the Atlantic Ocean revealed that M and A2 superfamilies appeared to be more expressed in the former whereas the O2 superfamily was more expressed in the latter.

## 1. Introduction

Cones are marine venomous snails that live in tropical and subtropical waters around the world hunting on worms, snails, and fish [1]. The venom of cones is produced exclusively in a long tubular duct, the venom gland, and it is a cocktail composed of (i) hundreds of different short peptides termed conotoxins, which block the prey neuromuscular channel/receptors and (ii) hormones, which interfere with the prey transductional signals [2,3,4]. Each conotoxin is initially synthesized as a precursor, which includes, from the N- to the C-terminus, a hydrophobic signal domain (relatively highly conserved and used for classifying precursors into different superfamilies [5]), a propeptide region, and the cysteine-rich mature (functional) peptide [3,4]. During the synthesis, the precursor is cleaved into the three domains, and disulfide bonds are formed in the mature conotoxin, which is folded and postranslationally modified [6]. Each of the >900 species of cones produces its own (unique) repertoire of conotoxins, which can vary among individuals [7,8,9] but also within the same individual depending on its physiological condition [10] or on whether venom is used to capture prey or as a defense against predators [11]. Therefore, one of the main challenges in cone venomics is cataloguing the exact composition of the conotoxin toolkit of each of the species [7,8,9,12]. This is important for evolutionary studies, as comparing species venom repertoires within a phylogenetic framework provides insights on how their diversity was generated, their adaptation to different diet specializations, and their relative influence in the extraordinary species diversification of the group [8,13,14,15,16]. Moreover, the enormous conotoxin diversity available in nature is considered a potentially valuable source of novel drugs to advance in neuroscience research [17,18,19], as well as to treat human neuropathology disorders and clinical complications [20,21]. 

For many years, mature conotoxins were purified from secreted venoms using different chromatographic methodologies, their amino acids were sequenced, and their physiological roles were characterized (e.g., [22]). This was a very helpful approach for detecting medically valuable individual conotoxins, but made cumbersome the task of identifying the full (precursor) conotoxin toolkit of a given cone species. In more recent years, the advent of massive RNA sequencing (RNA-seq) techniques has revolutionized the pace of cone venom cataloguing. Currently, it is becoming a standard practice to extract the messenger RNAs expressed in the venom duct of an individual, to sequence the whole transcriptome, and to identify all conotoxin precursor transcripts by comparison against curated reference databases [23,24,25]. The technique has been shown to be highly efficient as even rare transcripts are detected, but necessary controls on the quality and coverage of the assembled contigs need to be implemented to avoid potential artifacts that may overestimate conotoxin sequence diversity [7]. Moreover, analyzing venom gland transcriptomes in several individuals per species is useful for quantifying the differential expression of conotoxins [7] and for identifying peptide peaks obtained through advanced mass-spectrometry techniques directly from the venom [26,27].

To date, the list of sequenced venom gland transcriptomes has grown, steadily improving our knowledge of the vast diversity of conotoxins produced by different cone species such as, e.g., *Chelyconus ermineus* [7], *Cylinder gloriamaris* [28] or *Dendroconus betulinus* [9], just to give examples of cones that hunt on fishes, snails, and worms, respectively. One notable exception to this list is the magician’s cone, *Pionoconus magus* (Linnaeus, 1758), one of the earliest described and most well-known cone species [29]. Although the venom gland transcriptome of one specimen of *P. magus* from the Philippines was recently sequenced, it was used (along with those of other cone species) to test the performance of a new method for identifying highly divergent conotoxin precursors using machine learning algorithms, and not to describe in detail the venom repertoire of this emblematic species [30]. This is even more surprising knowing that the only conotoxin approved for medical use (as pain reliever) is the ω-conotoxin MVIIA, an antagonist of calcium channels, known commercially as Ziconotide or Prialt, which was isolated from *P. magus* [31,32]. The magician’s cone is a medium to large-size cone (16–94 mm) distributed throughout the Indo-Pacific region, although showing great variability in shape and color patterns of the shell between different populations [33]. The protoconch is paucispiral [34] and eggs are large (>550 µm) [35], both features indicating a non-planktotrophic larval development. While juveniles were reported to have vermivorous-like radular teeth and to feed on syllid polychaetes [36], the adults are piscivorous, have a modified radular tooth, and use a ‘taser-and-tether’ strategy for hunting [37]. Thus far, a total of 18 conotoxin precursors have been reported for *P. magus*, belonging to the superfamilies A, O1, and O2 as retrieved from ConoServer [38] on July, 2019 (http://www.conoserver.org/index.php?page=list&table=protein&Organism_search[]=Conus%20magus&Type[]=Precursor). Six of these precursors corresponded to conotoxins isolated from the milked venom of a Northern Australian individual [39]. Also, comparative analyses of the venom gland transcriptome of one individual of *P. magus* from the Philippines using similarity searches reported 49 canonical conotoxin precursors and about 950 additional highly divergent candidates identified using machine learning methodologies [30]. The magician’s cone is the type species of the genus *Pionoconus* [34]. Within this genus, venom gland transcriptomes are also available for *P. catus* [26] and *P. consors* [40], Expressed Sequence Tags (ESTs) for *P. striatus* [41], and the evolution of the A conotoxin superfamily was studied in detail in several species [42].

Here, we sequenced and analyzed the venom gland transcriptomes of two individuals of *P. magus* from the Okinawa archipelago (Japan). We annotated all conotoxin precursors found in these two individuals as well as those found in the one reported from the Philippines [30] using similarity searches. Most precursors were assigned to known superfamilies but two putative new superfamilies were also identified based on their divergent signal domains. We compared the composition of the venoms of the three individuals in terms of number and diversity of superfamilies as well as determined differential expression patterns against *Chelyconus ermineus*, the only piscivorous cone species in the Atlantic Ocean [7].

## 2. Results and Discussion 

### 2.1. Quality of the Assembly 

The transcriptomes of the venom ducts of two specimens of *P. magus* from Okinawa (Japan) were sequenced. A total of 30–40 million raw reads were produced per sample (Table 1). After quality filtering, 98% of the reads remained, and 60,000–70,000 contigs were assembled per individual (Table 1). In addition, the transcriptome of the venom gland of an individual of *P. magus* from the Philippines was assembled from raw reads available in the SRA database (SRX5015024) [30]. This latter transcriptome was based on about half the number of raw reads but had almost double the number of contigs (Table 1). The completeness of the assemblies was assessed using two alternative metrics. Transrate assembly scores for all individuals were 0.3 (Table 1). The two individuals from Okinawa retrieved 34% of the metazoan conserved single-copy orthologs (BUSCO) whereas that from the Philippines retrieved up to 72%. While de novo assemblies combining RNA from several tissues and/or different developmental stages tend to have higher BUSCO scores [43] as that obtained for the Philippines specimen, the expected values for transcriptomes derived from specialized tissues such as the venom gland are more in agreement with the results obtained for the Okinawa samples. For instance, the obtained levels of BUSCO completeness for Okinawa specimens and Transrate assembly scores for all samples were similar to those reported for the venom glands of snakes and scorpions [44]. Up to 34% of clean reads mapped onto contigs encoding for conotoxin precursors and hormones, indicating that an important fraction of the transcription in the venom gland is devoted to venom production (43% was reported for *P. consors*, [40]; 57% was reported for *C. ermineus* [7]). 

### 2.2. Diversity of Conotoxin Precursors and Hormones in P. magus

**Figure 1:**
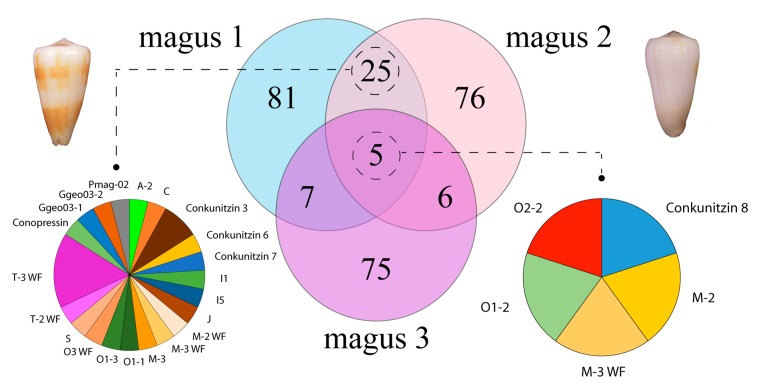
Venn diagram representation of conotoxin precursor and hormone sequences that were shared or unique to the two *P. magus* individuals from Okinawa (dorsal views shown) and that from the Philippines. For the shared sequences, the proportions of the different conotoxin superfamilies are shown.

Annotation of the transcripts expressed in the venom duct using BLASTX searches against a curated reference database rendered 118, 112, and 93 distinct (with at least one amino acid difference) conotoxin precursor and hormone sequences in magus1, magus2, and magus3 individuals, respectively (Figure 1). Only five conotoxin precursors were present in all specimens (Figure 1). The two individuals from Okinawa shared 30 conotoxin precursors and hormones. The total number of conotoxin precursors and hormones per individual as well as the high observed intraspecific diversity are similar to those reported for different cone species. For example, 91–98 (38% shared) and 145–176 (20% shared) conotoxin precursors and hormones were found for three individuals of *D. betulinus* [9] and three individuals of *C. ermineus* [7], respectively. The low number of shared sequences between the individual from the Philippines and those from Okinawa may be related to isolation by distance processes [45], in agreement with the high phenotypic variability described for *P. magus* [33] and could reflect the existence of a species complex. However, analyzed individuals showed low sequence divergences in complete mitochondrial genomes (Supplementary Figure S1). Alternatively, differences in venom gland transcriptome composition could reflect diet adaptations to hunt on local preys, as reported for the populations of *Virroconus ebraeus* in Okinawa, Hawaii, and Guam [46]. However, the two *P. magus* individuals from Okinawa were from nearby populations within the same island, thus other factors than geographic distance such as differences in age/size [9,47] or physiological condition [10] could be responsible for the observed intraspecific variation in precursor expression. 

A total of 12 out of the 18 previously reported conotoxin precursors and hormones from *P. magus* available at ConoServer were recovered corresponding to different members of A and O1 superfamilies (Supplementary File S1). Of these, six corresponded to α- and ω-conotoxins isolated from the milked venom of captive *P. magus* from Night Island in northern Australia [39]. Four of them (except α-conotoxins MIC and MI) were found in the Philippines individual, and only two (α-conotoxin MII and ω-conotoxin MVIIB) in the Okinawa individuals. This pattern suggests simplified milked venoms compared to transcriptomes and a higher similarity in the venom composition (at least for the two analyzed superfamilies) between the individuals from Australia and the Philippines compared to Okinawa specimens. In any case, >80% of the milked venom remained uncharacterized in the original study [39], which could account for at least some of the conotoxins identified by our transcriptomic approach. Moreover, the high correspondence between proteomic and transcriptomic data in *P. magus* further supports the validity and complementarity of both methodologies. We also recovered 24 out of the 49 conotoxin precursors identified through similarity searches in the original study of the individual from the Philippines [30]. Interestingly, the ω-conotoxin MVIIA (Ziconotide) was only found in the Philippines individual, although it had not been reported in the original transcriptomic study [30]. These results suggest that ω-conotoxin MVIIA might not be an essential component of the *P. magus* venom for fish hunting as the Okinawa specimens express other O1 superfamily paralogs instead as MVIIB (Supplementary File S1) [39]. Nevertheless, transcriptomic and proteomic data from further individuals would be needed to confirm this hypothesis. Altogether, these results indicate that due to the intrinsic variability of conotoxins, retrieving the whole conotoxin repertoire of a transcriptome is highly dependent, on one hand, on the assembly procedure and, on the other hand, in the geographical source of the samples in first instance [27] and secondarily on the physiological state, age, and other natural history traits. 

The total number of distinct precursors for *P. magus* was 275, of which 234 were assigned to 33 known conotoxin precursor superfamilies and 13 to six hormone families (conopressin-1, conopressin-2, insulin-2, insulin-5, prohormone-4, and Thyrostimulin hormone beta 5; Supplementary Table S1 and File S2). Moreover, we found 12 new members of six superfamilies that were recently described as new in *C. ermineus* (hereafter Cerm superfamilies; [7]), four additional members of one superfamily that was described anew in *Gastridium geographus* (hereafter Ggeo superfamily; [11]), three additional members of three superfamilies described anew in *Rhombiconus imperialis* (hereafter Rimp superfamily; [27]), two additional members of a superfamily found newly in *Rhizoconus miles* (hereafter Rmil superfamily; [48]), and one additional member of a superfamily found newly in *Turriconus andremenezi* (hereafter Tand superfamily; [8]), expanding further the taxonomic distribution of these recently reported superfamilies (Supplementary Table S1 and File S2). In addition, up to six putative conotoxin precursors that had orthologs in other cone species showed signal sequences distinct enough (<70% identity) from those of known superfamilies to propose two new superfamilies (hereafter Pmag superfamilies). The new conotoxin precursor superfamily Pmag01 was represented by a single sequence in individual magus2 and had a VI/VII cysteine framework, whereas Pmag02 was represented by five sequences expressed in all individuals and had a XXII cysteine framework. Functional studies will be needed to characterize the physiological and pharmacological properties of their mature peptides.

**Figure 2:**
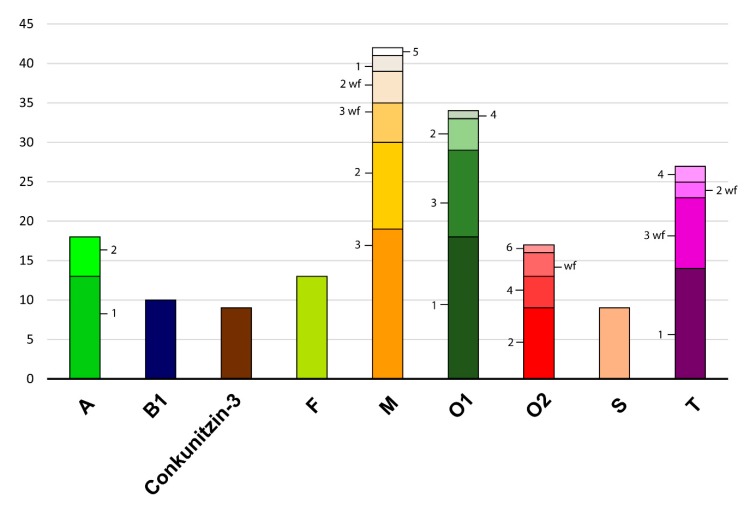
Histogram of the most diverse conotoxin precursor superfamilies found in *P. magus*. *Y* axis indicates number of different precursors and color shades indicate proposed paralogs (coded with numbers) within each superfamily.

Overall, the superfamilies that were represented by the highest number of conotoxin precursors were M (42), O1 (34), T (27), A (18), O2 (17), and F (13), accounting for 55% of the total diversity (Figure 2). All these superfamilies except F had several identical precursors in the two specimens from Okinawa. Members of the F superfamily were also present in the two individuals (but not in the one from the Philippines) although precursors were not identical (Figure 1). Other precursors common in the two individuals from Okinawa belonged to O3, conkunitzin, C, I, J, S, Ggeo03, and Pmag02 superfamilies (Figure 1). Notably, about one third of the members that were identical in the two specimens from Okinawa corresponded to mature conotoxins without cysteine frameworks (e.g., half of the M members, and all O3 and T members), indicating a slower rate of evolution of this type of conotoxins. The only precursors common to the three individuals were one member each of O1, O2, M-2, M-3 WF, and conkunitzin 8 superfamilies (Figure 1). The high diversity of M, O1, O2, and T superfamilies has been reported for many other cone species such as, e.g., the piscivorous *C. ermineus* [7], the molluscivorous *C. gloriamaris* [28], and the vermivorous *D. betulinus* [9]. Therefore, producing various members of these conotoxin superfamilies seems to be essential for the wider venom activity of cone snails regardless of their diet. The M superfamily includes µ-conotoxins that are antagonists of neuronal voltage-gated Na^+^ channels, κM-conotoxins that block voltage-gated K^+^ channels, and ψ-conotoxins that are non-competitive antagonists of nicotinic-acetylcholine receptors (nAChRs; [3]). The O1 superfamily includes μ-, κ- and ω-conotoxins, which are antagonists of voltage-gated Na^+^, K^+^, and Ca^2+^ channels, respectively, as well as δ-conotoxins, which block the inactivation of voltage-gated Na^+^ channels [3]. The O2 superfamily has γ-conotoxins that modulate Ca^2+^ (e.g., pacemaker) channels [3]. Several functions have been reported for the diverse members of the T superfamily but the exact targets for these conotoxins remain elusive, although it has been suggested that they could be antagonists of G protein-coupled receptors [3]. 

**Figure 3:**
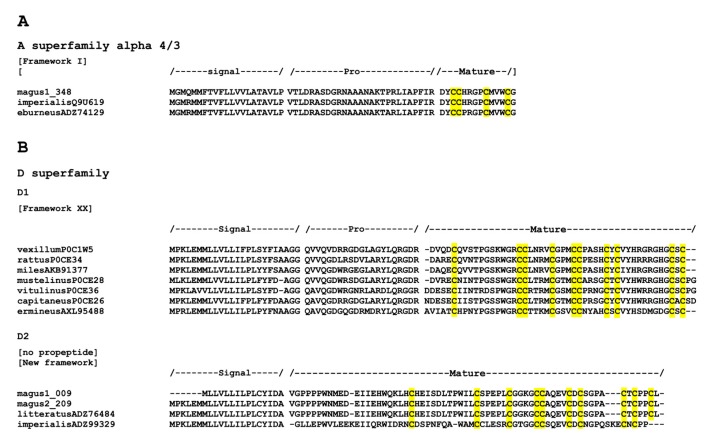
(**A**) The member of the A superfamily alpha 4/3 in *P. magus*; (**B**) two paralogs in the D superfamily. The signal, propeptide, and mature regions (with their cysteine patterns) are shown.

The high diversity of conotoxins within the A superfamily is typical of piscivorous cones such as *G. geographus* [49], *Textilia bullatus* [50], *C. ermineus* (with slightly less members but high expression levels; [7]), and species of the genus *Pionoconus* [42] including *P. catus* [26], *P. consors* [40], and *P. magus* (this paper). The diversity within the A superfamily can be subdivided into two groups: (i) kappa, which contains conotoxins targeting selectively K^+^ channels, producing an excitatory effect [3] and that are exclusive of piscivorous species; and (ii) alpha, which contains conotoxins that preferentially target nAChRs and ultimately inhibit neuromuscular transmission producing paralysis [51]. All cones species produce mature peptides of the alpha subfamily having the α4/7 cysteine spacing. In addition, piscivorous cone species from the Indo-Pacific region like *P. magus* produce mature peptides with the α3/5 cysteine spacing. Instead, the only piscivorous cone species of the Atlantic Ocean, *C. ermineus* exhibits high expression of mature peptides with the α4/4 cysteine spacing. This distinct pattern together with the distant relative phylogenetic position of *C. ermineus* with respect to Indo-Pacific piscivorous cones support a convergent evolution of fish hunting in cones [7]. Furthermore, until recently, the α4/3 cysteine spacing was almost exclusively found in the alpha conotoxins of the genus *Rhombiconus* [52], whose species are specialized in hunting fireworms [53]. However, this conotoxin has now been found in *P. magus* (Figure 3).

The F superfamily, whose function remains unknown [3], shows a high diversity in one of the analyzed specimens of *P. magus* but neither in the other studied individuals nor in other cone species. Other conotoxin superfamilies have also display experienced bursts of diversification like P superfamily in *Turriconus* [8] and d in *Rhizoconus vexillum* [54]. The evolutionary significance of these species-specific superfamily expansions and why some individuals and not others show them within the same species remains unclear to date.

### 2.3. Diversity within Superfamilies

**Figure 4:**
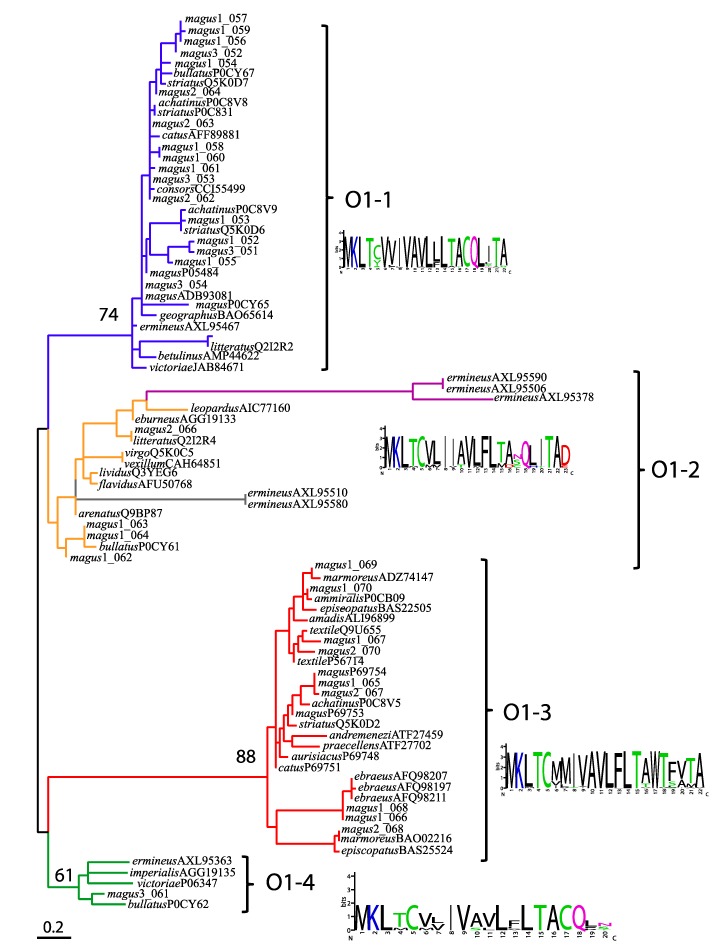
Phylogeny of the O1 superfamily. A maximum likelihood tree using the signal + propeptide regions was reconstructed. Paralog groups are indicated in different colors and the consensus signal sequence for each paralog group is shown. Bootstrap values of main clades are indicated except for O1-2, which was below 50%. Scale bar indicates expected substitutions/site. GenBank accession numbers are indicated after each species except for *P. magus*.

The large number of members assigned to A, M, O1, and T superfamilies could be further classified into distinct paralogs when taking into consideration both sequence similarity in the propeptide and mature domains as well as the potential presence of different cysteine patterns (Supplementary File S2). For instance, in a previous study, up to five groups were proposed within the M superfamily taking into account the number of residues present in the third intercysteine loop [55]. Phylogenetic analysis of the diversity within A superfamily within *Pionoconus* delimited at least four paralog groups differing in their cysteine patterns (alpha versus kappa) and in the spacing between cysteines in the alpha group (4/3, 4/4, and 4/7; [42]). Phylogenetic analyses of the diversity within the M and T superfamilies of different cone species distinguished four and three paralog groups, respectively and provide insights onto the evolutionary origins of mature peptides lacking cysteines [7]. Here, we reconstructed the phylogeny of the O1 superfamily based on signal plus propeptide alignments and classified the conotoxins precursors of *P. magus* into four paralogs (Figure 4). Notably, all the O1 precursors shared a conserved first half of the signal domain (MKLTC motif; [3]) but the different paralog groups varied in the second half of the signal domain and the propeptide region, which are likely the one having the residues that define the clades in the reconstructed phylogenetic tree (Figure 4; Supplementary File S2). Moreover, despite all O1 precursors shared the VI/VII cysteine framework in the mature peptide, each paralog group contained members that have been associated to distinct physiological activities: O1-1 has ω- and κ-conotoxins, O1-2 has µ-conotoxins, and O1-3 and O1-4 have δ-conotoxins [3]. The paralogs that were found in more cone species were O1-1 and O1-3 whereas paralogs O1-5 and O1-6 were exclusive of *C. ermineus* [7] and showed extremely long branches within paralog O1-2, which may indicate that they are just highly divergent members of this latter paralog (Figure 4).

Among less diverse superfamilies, the presence of a member of the D superfamily is remarkable (Figure 3). This superfamily was originally described in the Indo-Pacific vermivorous *R. vexillum* [56,57]. In this species, the mature peptide is unusually large [3]), has the XX cysteine framework, and was shown to be a nicotinic receptor antagonist [56]. The defensive venom of *R. vexillum* is produced in the proximal region of the venom duct and it is almost exclusively composed of d conotoxins [54]. This superfamily is also found in other species of the genus including *Rhizoconus capitaneus*, *Rhizoconus rattus*, *R. miles*, and *Rhizoconus mustelinus* [54,57], but also in *Strategoconus vitulinus*, *Strategoconus generalis* [58], and *Strategoconus planorbis* [59], the Eastern Pacific *Ductoconus princeps* [60] and most strikingly in the Atlantic piscivorous *C. ermineus* [7] (Figure 3). The member of the D superfamily in *P. magus* showed no propeptide region and a larger and highly divergent mature region compared to those of *R. vexillum*. Hence, it was assigned to a different paralog group (Figure 3). This paralog was also reported in *R. imperialis* [61] and found using BLAST searches against GenBank in *Elisaconus litteratus* (unpublished sequence). The mature peptide in this paralog has a new cysteine framework (C-C-C-CC-C-C-C-C-C). The function of this second paralog and whether it also has defensive role is unknown.

### 2.4. Conserved Conotoxin Precursors

Given the observed general high variability of conotoxin precursors even at the intraspecific level [7,9], it was a striking finding that some sequences belonging to A, D, K, M, O1, O2, P, S, T, and conantokin-F superfamilies were highly (or fully) conserved among distantly related species (Supplementary File S3). Several of these sequences matched orthologues in the closely related piscivorous genus *Pionoconus* but others had identical sequences in distantly related species such as the vermivorous *E. litteratus*, *Lithoconus leopardus*, *Conus marmoreus*, and *Darioconus episcopatus*; *R. imperialis* (which hunts fireworms), and the molluscivorous *Cylinder textile*. Moreover, in some cases the 100% sequence identity occurred not only at the amino acid level but also at the nucleotide level (Supplementary File S3). The presence of such conserved sequences may be due to convergence phenomena and purifying selection, which could indicate that these copies of the contoxin precursors/mature peptides might be particularly efficient and specific in their function. These conotoxins might be used in the different cone species as a defense from a common predator, or target very conserved receptors in different types of prey (from worms to fish).

### 2.5. Other Proteins Identified in the Venom Gland Transcriptome

Besides conotoxin precursor and hormone transcripts, the venom gland of *P. magus* expressed up to 98 transcripts (Supplementary File S2) corresponding to proteins involved in the processing of conotoxins like, e.g., protein disulfide isomerases [62] or in enhancing venom activity like, e.g., conoporins [23,63] as has been described for other cones [23,40,50] and ubiquitously for other venomous animals from cnidarians [64] to snakes [65]. Moreover, we found through TBLASTX searches against NCBI NR database that several proteins previously assigned to conotoxin superfamilies were originally defined based on wrong open reading frames (ORFs). This is the case of the R superfamily [66], which was described from a shifted ORF of the proteasome subunit alpha type 4. Actually, this latter protein has proteolytic activity and was found in the exudates generated in tissues injected with snake venom [67]. Other cases of shifted ORFs applied to Cerm 17 and 19 [7], and W and Z [66] superfamilies, which genuinely correspond to conserved hypothetical proteins of unknown function. Finally, it is interesting to note the detection of a transcription factor, which is highly conserved not only across animals but also found in vascular plants (Supplementary File S2).

### 2.6. Relative Transcript Abundance and Differential Expression Analysis 

**Figure 5:**
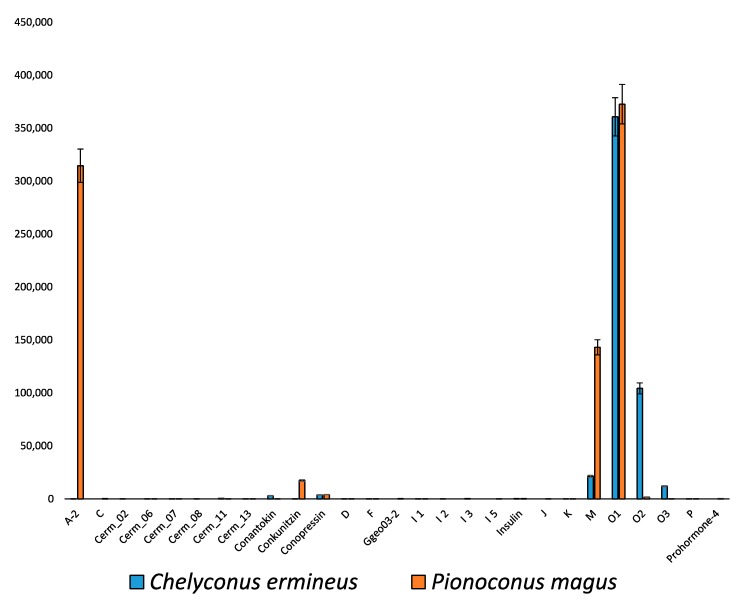
Differential expression between the Indo-Pacific *P. magus* and the Atlantic *C. ermineus* piscivore cone snails. *X* axis represents different high expressed superfamilies whereas *Y* axis is the average number of TPMs among individuals. Error bars represent standard error of the mean.

Analyses of the relative abundance of transcripts based on the number of raw reads showed that an O1-3 member (identified as MVIB according to its sequence) dominated in magus1 (48.6% of the total) but had much lower representation in the other individuals (Supplementary Figure S2). This is a δ-conotoxin, which blocks voltage-gated Na^+^ channels [68] and is a key component of the lightning-strike cabal. The A-2 superfamily conotoxins were the most abundant in magus2 (53.3%; Supplementary Figure S2). These conotoxins show the IV cysteine framework and were found to be K^+^ channel blockers in the closely related *Pionoconus striatus* [69]. Finally, the most abundant conotoxin in magus3 was an A superfamily alpha 3/5 (15.6%), although members of the M and O1 superfamilies were also abundant (Supplementary Figure S2). The alpha 3/5 inhibits muscle nAChRs and has been shown to be key in fish hunting in piscivorous Indo-Pacific cones [70,71]. With regards to the differential expression analyses between the piscivorous Indo-Pacific *P. magus* and the Atlantic *C. ermineus*, the M and A-2 superfamilies appeared to be more expressed in the former whereas the O2 and O3 superfamilies were more expressed in the latter. Both species showed high levels of expression of the O1 superfamily (Figure 5).

## 3. Materials and Methods

### 3.1. Sampling and RNA Extraction

We analyzed individuals OK194 (magus1) and OK206 (magus2) of *P. magus* from Japan, collected in Ishigaki Island in 2017 (Table 1). Their species identity was first determined based on the morphology of the shell and then further corroborated with a maximum likelihood phylogenetic analysis using mitochondrial genomes of different cone genera (Supplementary Figure S1) and methods described in [72].

Each individual, in a resting stage, was extracted from the shell and dissected to remove the complete venom duct, which was stored in 1 mL RNAlater (Thermo Fisher Scientific, Waltham, MA, USA) at −20 °C. For RNA extraction, each venom duct was grinded in 300 µL of TRIzol (Thermo Fisher Scientific, Waltham, MA, USA) and mixed with 60 µL of chloroform. After centrifugation, the aqueous phase was recovered and RNA precipitated in one volume of isopropanol and incubated overnight at −80 °C. The Direct-zol RNA miniprep kit (Zymo Research, Irvine, CA, USA) was used to purify total RNA (5–15 µg) following manufacturer’s instructions. 

### 3.2. Library Preparation and Sequencing 

Dual-indexed cDNA libraries (307–345 bp insert average size) for each sample were constructed after isolation of mRNA using the TruSeq RNA Library Prep Kit v2 (Illumina, San Diego, CA, USA) and following the manufacturer’s instructions. The quality of the libraries was analyzed with the 4200 TapeStation and the High Sensitivity D1000 ScreenTape assay (Agilent Technologies Inc., Santa Clara, CA, USA); libraries were quantified using real-time PCR in a LightCycler 480 (Roche Molecular Systems Inc., Pleasanton, CA, USA). The pool of indexed libraries (including samples from other projects) was loaded into different lanes and sequenced by paired-end sequencing (2 × 100 bp) in an Illumina HiSeq2500 (two flow cells) following standard procedures at Sistemas Genómicos (Valencia, Spain).

### 3.3. Transcriptome Assembly

Following [7], the reads corresponding to the two individuals were sorted using the corresponding library indices. Here, we added to the pipeline also the analysis of raw reads from a third individual (magus3) from the Philippines [30]. The quality of the raw reads was checked using FastQC v.0.10.1 [73]. Transcriptome assembly was performed using Trinity v.2.6.6 [74] and default settings (minimum contig length: 200; sequence identity threshold: 0.95), after filtering and quality-trimming reads in Trimmomatic v.0.36. The completeness of the assembly was assessed using BUSCO v.3 (metazoa_odb9 dataset; [75]) and Transrate v1.0.3 [76]. The raw reads have been deposited at the NCBI SRA database (see accession numbers in Table 1).

### 3.4. Prediction and Annotation of Conotoxin Precursors and Associated Proteins

The amino acid sequences of all conotoxin precursors and other proteins produced in the venom gland of cone species available in GenBank release 226 [77], Uniprot release 2018_07 [78], and ConoServer release 06-08-2018 [38] were downloaded in 6 August 2018 to construct a custom reference database. Redundant (identical in sequence) entries were removed. Subsequently, putative conotoxin precursors and associated proteins were identified by BLASTX similarity searches of the assembled contigs against the reference database (E-value of 1 × 10^−5^). Selected sequences were translated into amino acids using the universal genetic code. TBLASTX similarity searches against the GenBank NR database and manual inspection were performed in order to discard false positives (hits not corresponding to canonical conotoxins due to wrong ORF selection) or assembly artifacts (in low coverage terminal positions and chimaeras). Duplicate and highly truncated (>55% of the estimated total length) peptide sequences were removed to produce the final working list of conotoxin precursors and associated proteins of the three individuals of *P. magus* (provided in Supplementary Table S1). The three (signal, propeptide, and mature) domains of the predicted conotoxin precursors and the cysteine frameworks of the mature functional peptides were identified using the Conoprec tool [38]. Assignment of precursors to different protein superfamilies (Supplementary File S2) was based on the two highest scoring full-length conotoxin precursor hits in the BLAST results as well as taking into account the percentage of sequence identity (>70%) to the highly conserved signal region [3,23] and a ML reconstructed tree using the signal region (not shown). Further refinement of the superfamily assignment and within classification into paralog groups was achieved by considering sequence similarity in the propeptide regions and common cysteine frameworks. All *P. magus* conotoxin precursor sequences were deposited (as nucleotide sequences) in GenBank under accession numbers (MN517272–MN517536, BK011195–BK011285).

### 3.5. Phylogenetic Analyses of the O1 Superfamily

We performed a phylogenetic analysis of the highly diverse O1 conotoxin precursor superfamily using the signal and propeptide domains. Multiple sequence alignment was performed with mafft v7 [79] using the L-INS-i option and default parameters. Phylogenetic relationships were inferred using maximum likelihood [80] with PhyML v.1.3.13 [81] under best-fit models selected by AICc. Statistical support was assessed with 1000 non-parametric bootstrap pseudoreplicates (BP).

### 3.6. Transcript Abundance and Differential Expression Analyses

Clean reads were mapped onto the assembled precursors and transcript abundance was calculated in transcripts per million (TPM), a metric which normalizes for gene length and sequencing depth. We run RSEM within Trinity v.2.6.6, which internally uses Bowtie2 [82]. TPM estimates were treated as a proxy to relative expression levels. In addition, to detect differential expression between the three individuals of *P. magus* and three individuals of *C. ermineus* [7] as biological replicates, we run the EBSeq software [83] as implemented in Trinity. The posterior probability of being differentially expressed (PPDE), setting the False Discovery Rate (FDR) at 0.05, of conotoxins as a whole and of each of the different superfamilies was estimated.

## 4. Conclusions

The composition of the venom repertoire of *P. magus* is dominated by high intraspecific variability, which increases with geographic distance. Yet, several conotoxin precursors of the most diverse superfamilies in *P. magus* are highly conserved at the amino acid and nucleotide levels not only compared to orthologs from other species of the genus *Pionoconus* but also to distantly related cone species. As on other cones, the most diverse superfamilies were M, T, O1, O2, and A. Particularly remarkable was the detection of precursors of the D superfamily (formerly associated to vermivorous cones) and of the A alpha 4/3 conotoxins (formerly associated to *Rhombiconus*). The detection of the ω-conotoxin MVIIA (Ziconotide) only in the Philippines individual may indicate that paradoxically its ecological role may not be essential for the cone despite its relevant clinical value for humans.

## Figures and Tables

**Table 1:** Sequencing and assembly statistics of the three specimens of *P. magus* analyzed in this work.

Sample	Specimen No.	Voucher ID MNCN	Location	SRA Accesion No.	Raw Reads	Clean Reads	Contigs	Conotoxin Reads	BUSCO	Transrate
magus1	OK194	15.05/87629	Ishigaki, Japan	SRR9831243	31,530,877	30,998,130	61.842	10,294,213	34.7%	0.33
magus2	OK206	15.05/87641	Ishigaki, Japan	SRR9831255	41,380,216	40,552,301	73.039	13,566,387	34.0%	0.30
magus3	–	–	Central Philippines	SRX5015024	16,303,626	15,925,034	129.180	4,045,608	72.4%	0.34

## Supplementary Materials

The following are available online at https://www.mdpi.com/1660-3397/17/10/553/s1, Supplementary Figure S1. Phylogeny of cone snails using mitogenomes; Supplementary File S1. Conotoxins and conotoxin precursors of *P. magus* from ConoServer detected in the transcriptomes; Supplementary File S2. Alignments of all conotoxins present in *P. magus*; Supplementary File S3. Highly conserved precursors across cone snails; Supplementary Table S1. List of conotoxins found in *P. magus*; Supplementary Figure S2. Relative abundance of transcripts based on raw reads in the three individuals.
