# Supplementary material for: Conotoxin Diversity in the Venom Gland Transcriptome of the Magician’s Cone, Pionoconus magus
Source: Mar Drugs. 2019 Sep 27;17(10):553. doi: 10.3390/md17100553 (PMC6835573; doi:10.3390/md17100553)
Supplement: Supplementary file 1 [file marinedrugs-17-00553-s001.zip › Supplementary Material/Suppl Fig S1.pptx]

## Slide 1
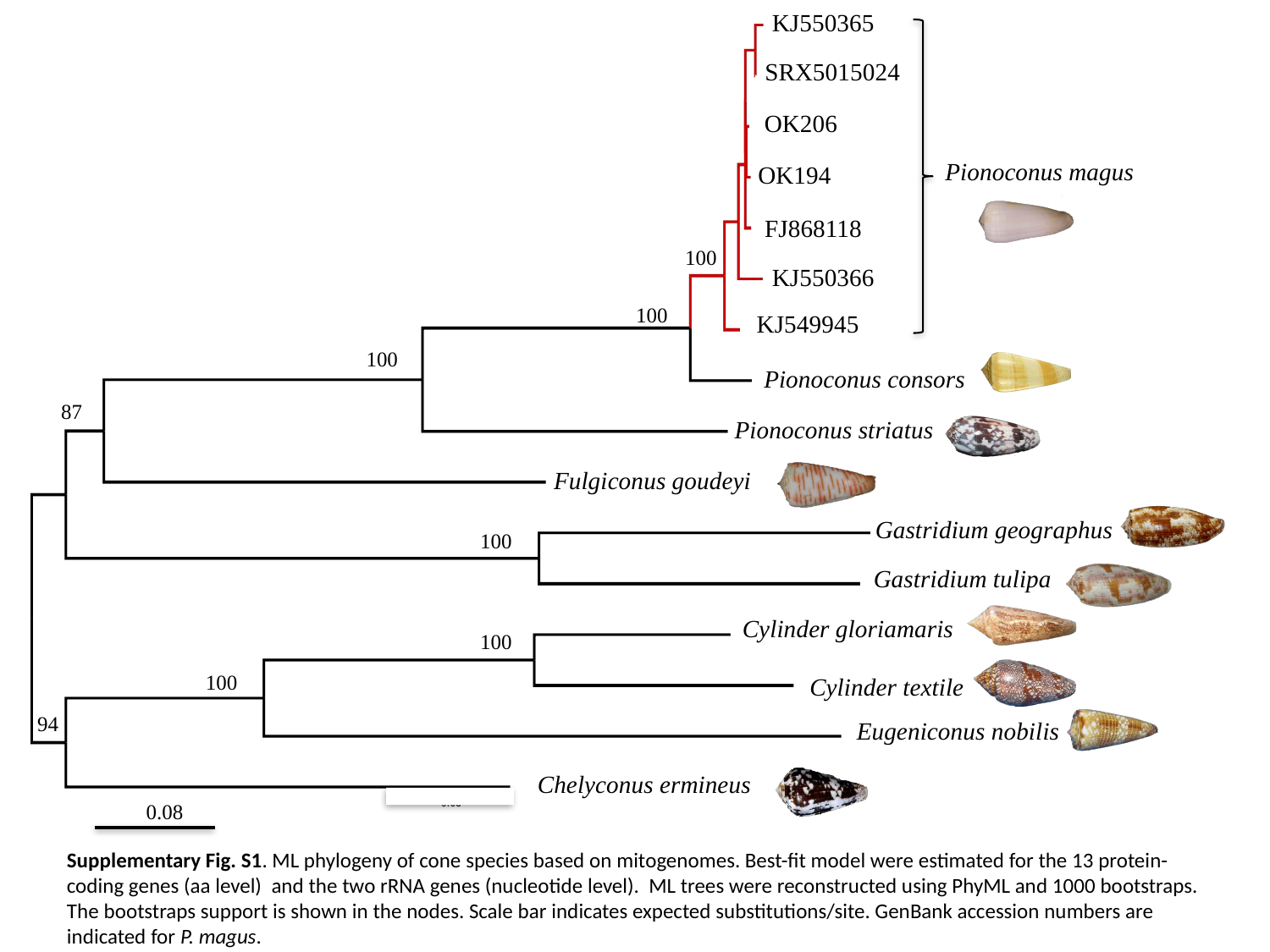

KJ550365
SRX5015024
OK206
Pionoconus magus
OK194
FJ868118
100
KJ550366
100
KJ549945
100
Pionoconus consors
87
Pionoconus striatus
Fulgiconus goudeyi
Gastridium geographus
100
Gastridium tulipa
Cylinder gloriamaris
100
100
Cylinder textile
94
Eugeniconus nobilis
Chelyconus ermineus
0.08
Supplementary Fig. S1. ML phylogeny of cone species based on mitogenomes. Best-fit model were estimated for the 13 protein-coding genes (aa level) and the two rRNA genes (nucleotide level). ML trees were reconstructed using PhyML and 1000 bootstraps. The bootstraps support is shown in the nodes. Scale bar indicates expected substitutions/site. GenBank accession numbers are indicated for P. magus.
