# Supplementary material for: Conotoxin Diversity in the Venom Gland Transcriptome of the Magician’s Cone, Pionoconus magus
Source: Mar Drugs. 2019 Sep 27;17(10):553. doi: 10.3390/md17100553 (PMC6835573; doi:10.3390/md17100553)
Supplement: Supplementary file 1 [file marinedrugs-17-00553-s001.zip › Supplementary Material/Suppl Fig S2.pdf]

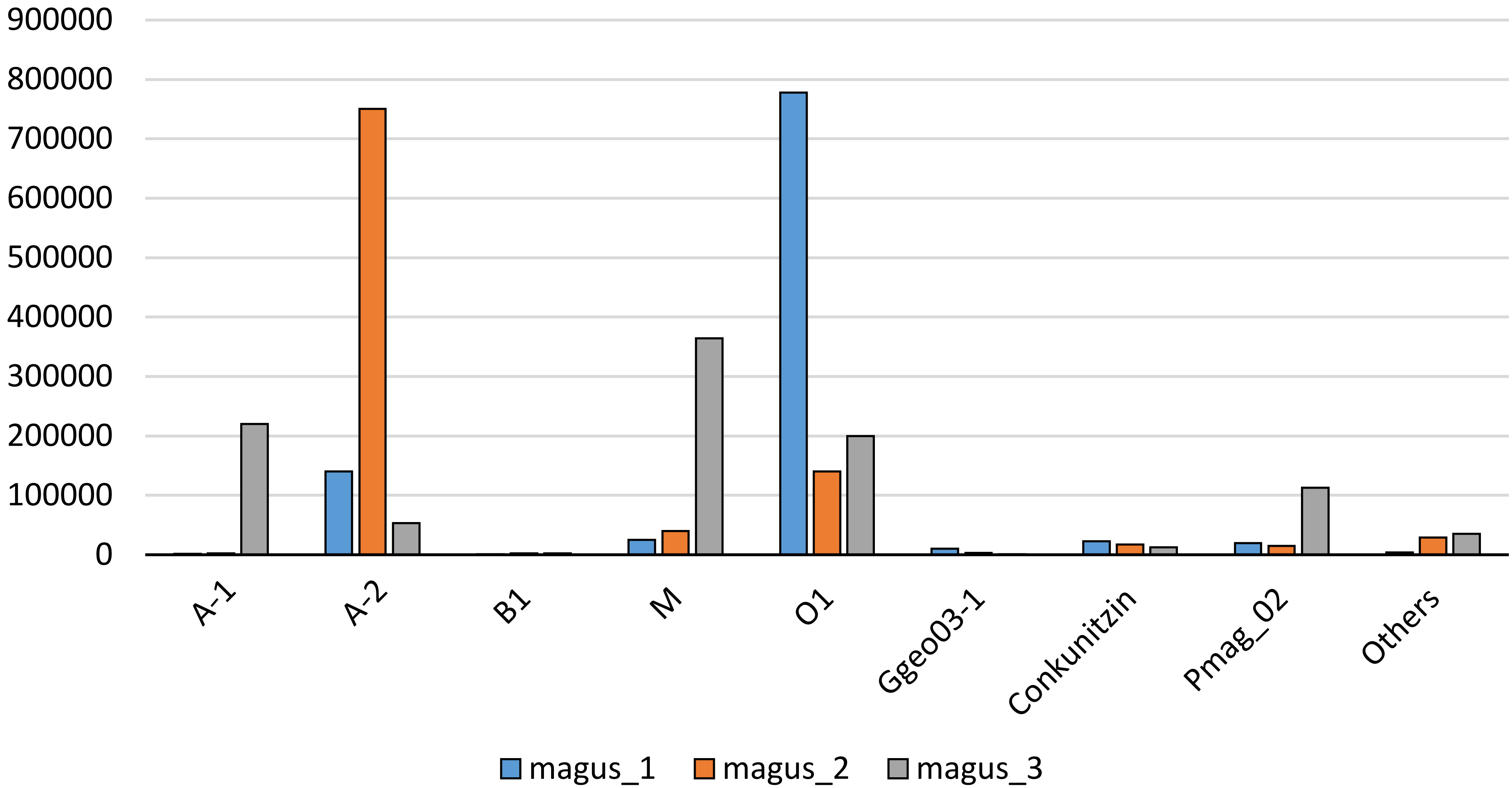

Supplementary Fig. S2. Relative abundance of transcripts. Clean reads were mapped onto the assembled CDS corresponding to conotoxin superfamilies and hormones of the three individuals. The read mapping was performed using Bowtie2 and TPM (Transcripts per million) values were calculated using the Trinity script "align_and_estimate_abundance.pl", with the RSEM algorithm.
