# Supplementary material for: Conotoxin Diversity in the Venom Gland Transcriptome of the Magician’s Cone, Pionoconus magus
Source: Mar Drugs. 2019 Sep 27;17(10):553. doi: 10.3390/md17100553 (PMC6835573; doi:10.3390/md17100553)
Supplement: Supplementary file 1 [file marinedrugs-17-00553-s001.zip › Supplementary Material/Suppl File S1.docx]

**Supplementary File S1. Conotoxins and conotoxin precursors of *P. magus* from ConoServer detected in the transcriptomes**

[Superfamily A-1]

[Framework I]

[alpha 3/5]

[ /------signal-------/------------Pro-------------/ CC---C-----C/-]

MIC [P05865] -------------------------------------------------- CCHPACGKNYSCX-

MI [P00023] ------------------------------------------------GR CCHPACGKNYSCX-

M1B [P00016] -----------------------------------------------NGR CCHPACARKYNCX-

M1.1 [P02999] -----MFTVFLLVVLTTTVVSFPSDRASDGRDDEAKDERSDMYESKRDGR CCHPACGQNYSCGR

magus3_003 ----------------TTVVSFPSDRASDGRDDEAKDERSDMYKSKRNGR CCHPACGKNYSCGR

M1A [P00014] -----------------------------------------------DGR CCHPACAKHFNCX-

magus1_004 MGMRMMFTVFLLVVLTTTVVSIPSDRASDGRDDEAKDERSDMYKSKRNGR CCHPACGKHFNCGR

magus2_007 MGMRMMFTVFLLVVLTTTVVSFPSDRASDGRDDEAKDERSDMYKSKRNGR CCHPACGRHFNCGR

magus3_004 MGMRMMFTVFLLVVLTTTVVSFPSDRASDGRDDEAKDERSDMYKSKRNGR CCHPACGKHFNCGR

[alpha 4/7]

[ /------signal-------/-----------Pro------------/ -CC----C-------C/---]

MII [P00008] MGMRMMFTVFLLVVLATTVVSFPSDRASDGRNAAANDKASDVITLALK GCCSNPVCHLEHSNLCGRRR

magus2_006 MGMQMMFTVFLLVVLATTVVSIPSDRASDGRNAAANDKASDVITLALK GCCSNPVCHLEHSNLCGRRR

magus3_002 IGMRMMFTVFLLVVLATTVVSFPSDRASDGRNAAANDKASDVITLALK GCCSNPVCHLEHSNLCGRRR

[Superfamily A-2]

[framework IV]

[Kappa 7/2/1/3]

[ /------signal-------/-------Pro------/ -----------CC-------C--C-C---C-------/----]

M4.2 [P03943] -----MFTVFLLVVLATTVVSIPSDRASDGRNAVVHER APELVVTATTTCCGFDPMTWCPPCMCTYSCSHQRKKPGRRND

magus1_006 MGMRMMFTVFLLVVLTTTVVSIPSDRASDGRNAVVHER APELVVTATTTCCGYDPMTWCPSCMCTYSCPHQRKKPGRRND

magus2_009 MGMQMMFTVFLLVVLATTVVSIPSDRASDGRNAVVHER APELVVTATTTCCGYDPMTWCPSCMCTYSCPHQRKKPGRRND

MIVA [P00018] MGMRMMFTVFLLVVLATTVVSIPSDRASDGRNAVVHER APELVVTATTNCCGYNPMTICPPCMCTYSCPPKR-KPGRRND

M4.3 [P03944] -----MFTVFLLVVLATSVVSIPSDRASDGGNAVVHER APELVVTATTTCCGYDPMTICPPCMCTHSCPPKG-KPGRRND

magus1_005 MGMQMMFTVFLLVVLATTVVSIPSDRASDGRNAVVHER APELVVTATTTCCGYDPMTICPPCMCTHSCPPKR-KPGRRND

magus2_008 MGMQMMFTVFLLVVLATTVVSIPSDRASDGRNAVVHER APELVVTATTTCCGYDPMTICPPCMCTHSCPPKR-KPGRRND

magus3_005 MGMQMMFTVFLLVVLATTVVSIPSDRASDGRNAVVHER APELVVTATTNCCGYNPMTICPPCMCTYSCPPKR-KPGRRND

[Superfamily O1]

[framework VI/VII]

[Omega/ Kappa]

[ /---------Signal-----/----------Pro---------/ C------C------CC---C-----C/]

MVIIA [P03999] MKLTCVVIVAVLLLTACQLITADDSRGTQKHRALRSTTKLSMSTR CKGKGAKCSRLMYDCCTGSCR-SGKCG

MVIIA [P01564] MKLTCVVIVAVLLLTACQLITADDSRGTQKHRALRSTTKLSTSTR CKGKGAKCSRLMYDCCTGSCR-SGKCG ziconotide

magus3_054 MKLTCVVIVAVLLLTACQLITADDSRGTQKHRALRSTTKLSMSTR CKGKGAKCSRLMYDCCTGSCR-SGKCG

MVIIB [P01638] --------------------------------------------- CKGKGASCHRTSYDCCTGSCN-RGKCX

magus1_061 -------IVAALLLTACQLITADDSRGTQKHRALRSDTKLSMSTR CKGKGASCHRTSYDCCTGSCN-RGKCG

magus2_063 MKLTCVVIVAVLLLTACQLITADDSRGTQKHRALRSDTKLSMSTR CKGKGASCHRTSYDCCTGSCN-RGKCG

magus3_055 MKLTCVVIVAVLLLTACQLITADDSRGTQKHRALRSDTKLSMSTR CKGKGASCHRTSYDCCTGSCN-RGKCG

MVIIC [P00822] -------------------------------------------TR CKGKGAPCRKTMYDCCSGSCGRRGKCG

magus1_058 MKLTYVVIVAVLLLTACQLITADDSRGTQKHRALKSDTKLSMSTR CKGKGAPCRKTMYDCCSGSCGRRGKCG

magus2_062 MKLTCVVIVAVLLLTACQLITADDSRGTQKHRALRSDTKLSMSTR CKGKGAPCRKTMYDCCSGSCGRRGKCG

MVIID [P00878] ------------------------------------------STR CQGRGASCRKTMYNCCSGSCN-RGRCG

magus3_053 MKLTCVVIVAVLLLTACQLITADDSRGTQKHRALRSDTKLSMSTR CQGRGASCRKTMYNCCSGSCN-RGRCG

[Superfamily O1-3]

[Delta]

[ /----Signal----------/------------Pro-------------/ --C------C------CC---C---C------/-----]

MVIB [P03079] MKLTCVMIVAVLFLTAWTFVTADDSRYGLKDLFPKERHEMKNPEASKLNQR EACYNAGSFCGIHPGLCCSEFCILWCITFVDSG------

magus1_065 -----------LFLTAWTFVTADDSRYGLKDLFPKERHEMKNPEASKLNQR EACYNAGSFCGIHPGLCCSEFCILWCITFVDSG------

magus2_067 MKLTCMMIVAVLFLTAWTFVTADDSRYGLKDLFPKERHEMKNPEASKLNQR EACYNAGSFCGIHPGLCCSEFCILWCITFVDSG------

magus3_059 --LTCMMIVAVLFLTAWTFVTADDSRYGLKNLFPKARHEMKNPEASK---- ---------------------------------------

MVIA [P03077] MKLTCVMIVAVLFLTTWTFVTADDSRYGLKNLFPKARHEMKNPEASKLNKR DGCYNAGTFCGIRPGLCCSEFCFLWCITFVDSG------

MVIC [P03078] MKLTCVMIVAVLFLTTWTFVTADDSRYGLKNLFPKARHEMKNPEASKLNKR DECYPPGTFCGIKPGLCCSAICLSFVCISFDF-------

MVID [P01621] --------------------------------------------------- EACYNAGTFCGIKPGLCCSAICLSFVCISFDF-------

M6.2 [P03081] MKLTCMMIVAVLFLTAWTFVTADDSRYGLKDLFPKERHEMKNPEASKLNQR EACYNAGTFCGIKPGLCCSAICLSFVCISFDLIDVFSSP

[others]

[ /----Signal----------/------------Pro-------------/ C------C-------CC---C---C--]

M6.1 [P03038] MKLTCVMIVAVLFLTVWTFATADDSGNGLEKLFSNAHHEMKNPEASKLNKR CKQADEPCDVFSLE-CCTGICLGFCTW

magus1_067 MKLTCMMIVAVFFLTVWTCATADDSGNGLEKLFSNAHHEMKNPEASKLNKR CKEADEPCSIFSLDRCCSGVCFGICMS

[ /--------Signal------/-----------Pro----------/ C------C-----CC---C------C-]

MgJ42 [P01112] MKLTCVLIIAVLFLTAYQLATAASHAKGKQKHRALRPADKHFRFTKR CNNRGGGCSQHPHCCSGTCNKTFGVCL

MgJr93 [P01127] MKLTCVLIIAVLSLTAYQLATAASHAKGKQKHRALRPADKHFRFTKR CNNRGGGCSQHPHCCSGTCNKIFGVCL

[ /-------Signal-----/--------------Pro-------------/ C------C-----CC---C-------C-]

MgJr94 [P01128] MKLTCVLIVVVLFLTACQLIPADYSRDTPGYPAWKLKTKMQNSRRWKLAKR CKGKGAGCDYSHECCSRQCTGRIFQTCN

[Superfamily O2]

[framework VI/VII]

[ /--------Signal-------/---------Pro---------/ -C------C-----CC----C----C-]

MgJr112 [P03639] MAKLTVLLLVAAVLLSTQVLVQGDGETPQRARFFTARKFSGVNKK GCDPKWTICNNDAECCFPYSCENSNCQ

[Conodipine-2]

[Framework new]

[ /---Signal---/---------------C------C--------C-------C-----C--C----------C-----------C-----

Conodipine [P05541] ---------------------QXPSTAELCKINSNACSVPFSXIPCQKXFLAACDRHDTCYHCGKHFGFKQDDCDDAFFRDMTALCAHGTD

Conodipine [P05540] -------------------------------------------------------------------------------------------

magus1_119 MKMLESALWILAALALPRIAAQNPSTAELCKINSNACSVPFSWIPCQKHFLAACDRHDTCYHCGKHFGFKQDDCDDAFFRDMTALCAHGTD

magus2_113 MKMLESALWILAALALPRIAAQNPSTAELCKINSNACSVPFSWIPCQKHFLAACDRHDTCYHCGKHFGFKQDDCDDAFFRDMTALCAHGTD

magus3_094 MKMLESALWILAALALPRIAAQDPSTAELCKINSNACSVPFSWIPCQKHFLAACDRHDTCYHCGKHFGFKQDDCDDAFFRDMTALCAHGTD

----C-----------------------------------------------C---------------------------C-----C---]

Conodipine [P05541] DEGXCP-------------------------------------------------------------------------------------

Conodipine [P05540] -------------------------------------------------AATCTHWALIYFKTVQLFGWXHFNYQVDATYCPQFQPCMP--

magus1_119 DEGSCPEKRKRREASSMSITTPLRQLRQLEKLVLPNSLSARDPRQLHSRAATCTHWALIYFKTVQLFGWWHFNYQVDATYCPQFQPCMPQV

magus2_113 DEGSCPEKRKRREASSMSITTPLRQLRQLEKLVLPNSLSARDPRQLHSRAATCTHWALIYFKTVQLFGWWHFNYQVDATYCPQFQPCMPQV

magus3_094 DEGSCPEKRKRREASSMSITTPLRQLRQLEKLVLPNSLSARDPRQLHSRAATCTHWALIYFKTVQLFGWWHFNYQVDATYCPQFQPCMPQV

[Superfamily I1]

[Framework XI]

[ /---Signal--------/---------Pro--------/ ----C------C-----CC-CC----C----------C--------]

M11.1a [P01420] ---------------------------------------- GAVPCGKDGRQCRNHADCCNCCPIGTCAPSTNWILPGCSTGQFMTR

M11.5 [P02807] ---------------------------------------- GHVPCGKDGRKCGYHADCCNCCLSGICKPSTSWTGCSTSTFD----

[ /---Signal--------/---------Pro--------/ ----C------C-----CC---CC----C------C-]

M11.2 [P02803] ---------------------------------------- ---TCSNKGQQCGDDSDCCWHLCCVNNKCAHLILLCNL

[Mu]

MIIIA [P01691] QGCCNVPNGCSGRWCRDHAQCCX
