# Supplementary material for: Conotoxin Diversity in the Venom Gland Transcriptome of the Magician’s Cone, Pionoconus magus
Source: Mar Drugs. 2019 Sep 27;17(10):553. doi: 10.3390/md17100553 (PMC6835573; doi:10.3390/md17100553)
Supplement: Supplementary file 1 [file marinedrugs-17-00553-s001.zip › Supplementary Material/Suppl File S3.docx]

**Supplementary Fig. S3. Conotoxin precursors highly conserved in different cone species**

[Superfamily A-1]

[alpha 4/3]

magus1_002 MGMQMMFTVFLLVVLATAVLPVTLDRASDGRNAAANAKTPRLIAPFIRDYCCHRGPCMVWCG

imperialisQ9U619 MGMRMMFTVFLLVVLATAVLPVTLDRASDGRNAAANAKTPRLIAPFIRDYCCHRGPCMVWCG

eburneusADZ74129 MGMRMMFTVFLLVVLATAVLPVTLDRASDGRNAAANAKTARLIAPFIRDYCCPRGPCMVWCG

[Superfamily A-2]

magus1_005 MGMQMMFTVFLLVVLATTVVSIPSDRASDGRNAVVHERAPELVVTATTTCCGYDPMTICPPCMCTHSCPPKRKPGRRND

magus2_008 MGMQMMFTVFLLVVLATTVVSIPSDRASDGRNAVVHERAPELVVTATTTCCGYDPMTICPPCMCTHSCPPKRKPGRRND

magus3_005 MGMQMMFTVFLLVVLATTVVSIPSDRASDGRNAVVHERAPELVVTATTNCCGYNPMTICPPCMCTYSCPPKRKPGRRND

consorsACZ49768 -----MFTVFLLVVLATTVVSIPSDRASEGRNAVVHERAPELVVTATTTCCGYDPMTICPPCMCTHSCPPKRKPGRRND

[Conantokin F]

magus2_016 MQSFTCMCLLVPLLLFFHLTQVSDTADHGGAATDVRSADRTLKRFPHDFRRAPKRRSDGHYDRNRKTSVQLDTELLVEGVREGQEERAEASYEKLLEIGR

eburneusADZ74131 MQSFTCMCLLVPLLLFFHLTQVSDTADHGGAATDVRSADRTLKRFPHDFRRAPKRRSDGHYDRNRKTSVQLDTELLVEGVREGQEERAEASYEKLLEIGR

magus2_016

eburneusADZ74131

[Superfamily D]

magus1_022 -----LMLLVLLILPLCYIDAVGPPPPWNMEDEIIEHWQKLHCHEISDLTPWILCSPEPLCGGKGCCAQEVCDCSGPACTCPPCL

magus2_026 MPKLEMMLLVLLILPLCYIDAVGPPPPWNMEDEIIEHWQKLHCHEISDLTPWILCSPEPLCGGKGCCAQEVCDCSGPACTCPPCL

litteratusADZ76484 MPKLEMMLLVLLILPLCYIDAVGPPPPWNMEDEIIEHWQKLHCHEISDLTPWILCSPEPLCGGKGCCAQEVCDCSGPACTCPPCL

magus1_022

magus2_026

litteratusADZ76484

[Superfamily K]

magus1_032 MIMRMTLTLFVLVVMTAASASGDALTEAKRIPYCGQTGAECYSWCIKQDLSKDWCCDFVKDIRMNPPADKCP

imperialisD0PX84 MIMRMTLTLFVLVVMTAASASGDALTEAKRIPYCGQTGAECYSWCIKQDLSKDWCCDFVKDIRMNPPADKCP

marmoreusAFE82855 ---------------TAASASGDALTEAKRIPYCGQTGAECYSWCIKQDLSKDWCCDFVKDIRMNPPADKCP

[Superfamily M-2]

magus2_050 -----GVLLTICLLLFPLTAVPLDGDQPADRPAERMQDDISSEQHPLFNQKRRCCRWPCPRYVDDEYCGCCLG

consorsCCI55490 -MSKLGVLLTICLLLFPLTALPMDGDQSVDRPAERMQDDISSEQHPLFNQKRRCCRWPCPRKIDGEYCGCCLG

striatusAEX60437 MMSKLGVLLTICLLLFPLTALPMDEDQSVDRPAERMQDDISSEQHPLFNQKRRCCRWPCPRYVDDEYCDCCLG

[Superfamily M-2-WF]

[conomarphin]

magus1_038 ---------------------------TADRHADQRGQDLTEQQRNSKRVLKKRDWEYHAHPKPNSFWTLV

magus2_052 -MSKLGVMLFIFLVLFPMATLQLDGDQTADRHADQRGQDLTEQQRNSKRVLKKRDWEYHAHPKPNSFWTLV

imperialisP0CH39 MMSKLGVLLCIFLVLFPMATLQLDGDQTADRHADQRGQDLTEQQRNSKRVLKKRDWEYHAHPKPNSFWTLV

marmoreusAGE10522 MMSKLGVLLCIFLVLFPMATLQLDGDQTADRHADQRGQDLTEQQRNSKRVLKKRDWEYHAHPKPNSFWTLV

[Superfamily M-3]

magus1_042 --LKMGVVLFIFLVLFPLATLQLDADQPVERYAENKQLLSPDERREIILHALGTRCCSWDVCDHPSCTCCSG

magus1_045 -MLKIGAVLFIFLVLFPLATLQLDADQPVERYAENKQLLNPYERREIILHALGTRCCSWDVCDHPSCTCCSG

gloriamarisGFNK01000030 -MLKMGVVLFIFLVLFPLATLQLDADQPVERYAENKQLLNPDERREIILHALGTRCCSWDVCDHPSCTCCGG

planorbisAEX60134 -MLKMGVMLFIFLVLFPLATLQLDADQPVERYAENKQLLSPDERREIILHALGTRCCSWDVCDHPSCTCCG-

textileAEX60127 MMLKMGVVLFIFLVLFPLATLQLDADQPVERYAENKQLLSPDERREIILHALGTRCCSWDVCDHPSCTCCG-

[Superfamily O1-1]

magus2_061 MKLTSVVIVAVLFLATCQLTTSDGSRGTWKDRAVRSITKVSMLRWPCKVAGSPCGLVSECCGTCNVLRNRCV

litteratusQ2I2R2 MKLTSVVIVAVLFLAACQLTTSDGSRGTWKDRAVRSITKVSMLRWPCKVAGSPCGLVSECCGTCNVLRNRCV

magus1_054 MKLTYVVIVAVLLLTACQLITADDSRGTQKHRALKSDTKLSMLTLRCASYGKPCGIYNDCCNTCDPARKTCT

magus1_056 MKLTYVVIVAVLLLTACQLITADDSRGTQKHRALGSKTKLSMLTLRCASYGKPCGIYNDCCNTCDPARKTCT

magus2_064 --------------------------GTQKHRALRSDTKLSMSTLRCASYGKPCGIYNDCCNTCDPARKTCT

magus3_052 MKLTCVVIVAVLLLTACQLITADDSRGTQKHRALGSKTKLSMLTLRCASYGKPCGIYNDCCNTCDPARKTCT

magus1_057 MKLTYVVIVAVLLLTACQLITADDSRGTQKHRALGSKTKLSMLTLRCASYGKPCGIQNDCCNACDPARKTWT

magus1_059 MKLTYVVIVAVLLLTACQLITADDSRGTQKHRALGSKSKLSMLTLRCASYGKPCGIQNDCCNACDPARKTWT

striatusQ5K0D7 MKLTCVVIVAVLLLTACQLITADDSRGTQEHRALRSDTKLSMLTLRCESYGKPCGIYNDCCNACDPAKKTCT

bullatusP0CY67 ---------AVLLLTACQLITAEDSRDTQKHRALRSDTKLSMLTLRCATYGKPCGIQNDCCNICDPARRTCT

magus1_053 MKLTYVVIVAVLLLTACQLITADDSRGTQKHRSLRSTTKVSKATDCIEAGNYCGPTVMKICC----------------

striatusQ5K0D6 MKLTCVVIVAVLLLTACQLITADDSRGTQKHRSLRSTTKVSKATDCIEAGNYCGPTVMKICCGFCSPYSKICMNYPKN

achatinusP0C8V9 MKLTCVVIVAVLLLTACQLLTADDSRGTQKHRSLRSTTKVSKATDCIEAGNYCGPTVMKICCGFCSPFSKICMNYPQN

[Superfamily O1-2]

magus2_066 MKLTCVLIIAVLFLMDNQLITADYPRDEQVYRAVRLRDAMQKSKGSGSCAYISEPCDILPCCPGLKCNEDFVPICL

litteratusQ2I2R4 MKLTCVLIIAVLFLMDNQLITADYPRDEQVYRAVRLRDAMQKSKGSGSCAYISEPCDILPCCPGLKCNEDFVPICL

eburneusAGG19133 MKLTCMVIIAVLFLMANQLITADYSRDEQVYRAVRLRDAMQKSKGSGSCADLSEACDILLCCPGLKCNEDFIPICL

[Superfamily O1-3]

magus1_069 MKLTCMMIVAVLFLTAWTFATADDPRNGLGNLFSKAHHEMKNPKDSKLNKRCLDAGEMCDLFNSKCCSGWCIILVCA

magus2_070 -----------------TFVTADDSGNGLENLFSKAHHEMKNPKDSKLNKRCLDAGEMCDLFNSKCCSGWCIILVCA

marmoreusADZ74147 MKLTCMMIVAVLFLTAWTFAAADDPRNGLENLFSKAHHEMKNPKDSKLNKRCLDAGEMCDLFNSKCCSGWCIILVCA

magus1_070 MKLTCMMIVAVLFLTAWTFATADDPRNGLGNLFSNAHHEMKNPEASKLNKRWCKQSGEMCNLLDQNCCEGYCIVLVCT

ammiralisP0CB09 MKLTCVMIVAVLFLTAWTFATADDPRNGLGNLFSNAHHEMKNPEASKLNKRWCKQSGEMCNLLDQNCCEGYCIVLVCT

textileQ9U655 MKLTCMMIVAVLFLTAWTFATADDSGNGLENLFSNAHHQMKNPEASKLNKRWCKQSGEMCNLLDQNCCDGYCIVLVCT

magus2_068 MKLTCMMIVAVLFLTAWTLVMADDSNNGLANHFLKSRDEMEDPEASKLEKRACSKKWEYCIVPILGFVYCCPGLICGPFVCV

marmoreusBAO02216 -----MMIVAVLFLTAWTLVMADDSNNGLANHFLKSRDEMEDPEASKLEKRACSKKWEYCIVPILGFVYCCPGLICGPFVCV

episcopatusBAS25524 MKLTCMMIIAVLFLTAWTFVMADDSNNGLANHFLKSRDEMEDPEASKLEKRDCQEKWEYCIVPILGFVYCCPGLICGPFVCV

magus1_066 MKLTCMMIVAVLFLTAWTSVTAGDFKNKLKNLSLKARKEVENPKASKLHQKRCVQTGGSCPSTTGCCNGLCNVDKCT

ebraeusAFQ98197 ----CILIVAVLFLTAWTSVTVGDFKNKLKNLSLKARKEVENPKASKLHQKRCVQTGGSCPSTTGCCNGLCNVDKCT

magus1_068 MKLTCMMIVAVLFLTAWTSVTAGDFKNKLKNLSLKARKEVENPKASKLHQKACINSGDPCQRTVRCCSRRCGVNSCA

ermineusAFQ98207 -----ILIVAVLFLTAWTSVTAGDFKNKLKNLSLKARKEVENPKASKLHQKACINSGDPCQRTVRCCSRRCGVNSCA

ebraeusAFQ98211 ----CILIVAVLFLTAWTSVTAGDFKNKLKNLSLKARKEVENPKASKLHQKACVNRGDPCQRAVRCCSRRCGVNSCA

[Superfamily O2-2]

(Contryphan)]

magus1_072 MQKLIILLLVAAVLMSTQAVLQEKRPKEKIKFLSKKKTDAEKQQKRLCPDYTEPCSHAHECCSWNCHNGHCTG

textileQ9BPB4 MQKLIILLLVAAVLMSAQAVLQEKRPKEKIKFLSKRKTDAEKQQKRLCPDYTEPCSHAHECCSWNCYNGHCTG

victoriaeG1AS83 MQKLIILLLVAAVLMSTQALFQEKRRKEKIDLLSKRKTDAEKQHKRLCPDYTDPCSNAYECCSWNCHNGHCTG

magus1_074 MEKLTILLLVAALLLSIQAVNQEKHQRAKINLLSKRKPPAERWWRWGGCMAWFGKCSKDSECCSNSCDITRCELMRFPPDW

textileQ9BHA0 MEKLTILLLVAAVLLSIQALNQEKHQRAKINLLSKRKPPAERWWRWGGCMAWFGLCSKDSECCSNSCDVTRCELMPFPPDW

victoriaeG1AS73 MEKLTILLLVAAVLMSIQAVNQEKHQRAKMNLLSKRKPPAERWWRWGGCMAWFGLCSKDSECCSNSCDVTRCELMPFPPDW

magus1_073 MEKLTILLLVAAVLMSTQALVERAGENRSKENIKFLLKRKRAADRGMWGDCKDGLTTCFAPSECCSEDCEGSCTMW

textileAAZ83753 MEKLTILLLVAAVLMSTQALVERAGENHSKENIKFLLKRKRAADRGMWGECKDGLTTCLAPSECCSEDCEGSCTMW

magus1_076 MEKLTILLLVTAVLMSTQALMQSGIEKRQRAKIKFFSKRKTTAERWWEGECYDWLRQCSSPAQCCSGNCGAHCKAW

imperialisAME17677 MEKLTILLLVTAVLMSTQALMQSGIEKRQRAKIKFFSKRKTTAERWWEGECYDWLRQCSSPAQCCSGNCGAHCKAW

magus1_076

imperialisAME17677

[Superfamily O2-6]

(Contryphan)]

magus1_079 MKLTILLLVAALLVLTQARTERRRVKSRKTSSTYDDEMATFCWSYWNEFQYSYPYTYVQPCLTLGKACTTNSDCCSKYCNTKMCKINWEG

imperialisAME17676 MKLTILLLVAALLVLTQARTERRRVKSRKTSSTYDDEMATFCWSYWNEFQYSYPYTYVQPCLTLGKACTTNSDCCSKYCNTKMCKINWEG

magus1_079

imperialisAME17676

[Superfamily P-1b]

magus2_078 MTLTKSAVLILVLLLAFDNFADVQPGLITMGGGRLSNLLSKRVSIWYCASRTCSAPADCNPCTCESGVCVDWL

litteratusABC74995 MTLTKSAVLILVLLLAFDNFADVQPGLITMGGGRLSNLLSKRVSIWFCASRTCSAPADCNPCTCESGVCVDWL

[Superfamily S]

magus1_086 MMSKMGAMFVLLLLFTLASSQQEGDVQARKTSLKSDFYRALRGCTLVNNCEKNGACNGDCHCKGKICKCGSSARPWKPGCACTCRNAK

magus2_080 MMSKMRAMFVLLLLFTLASSQQEGDVQARKTSLKSDFYRALRGCTLVNNCEKNGACNGDCHCKGKICKCGSSARPWKPGCACTCRNAK

consors(Terrat2012) -------MFVLLLLFTLASSQQEGDVQARKTRLKSDFYRAWRGCTLVNNCEKNGACNGDCHCKGKICRCSSSARPWKPGCACTCRNAK

magus1_088 MMSKMGAMFVLLLLFTLASSQQEGDVQARKIRLRNDFLRTSRMIFTRGCGGSCHTTPGCGGNCECNSPVPCYCSGTETCVCVCSG

imperialisAME17689 MMLKMGAMFAILLLFALSSSQQEGDVQARKIRLRNDFLRTSRMIFTRGCGGSCHTSPGCGGNCECNSPVPCYCSGTETCVCVCSG

[Superfamily T-1]

magus2_088 MRCLPVLIILLLLIPSAPSVDAQPMTKDDVPLASFHDNAKRTLKRLWNKRSCCPQEFLCCLYLVK

leopardusAAT01633 MRCVPVFIILLLLIPSAPSVDAQRKTKDDVPLASFHDNAKRTLKRLWNKRSCCPQEFLCCLYLVK

litteratusABC70187 MRCLPVFIILLLLIPSAPSVDAQRKTKDDVPLASFHDNAKRTLKRLWNKRSCCPQEFLCCLYLVK

magus2_082 MRCLPVFVILLLLIASAPSADARLKTKDDMPLPSSHANIKRTLQIHRNKRCCPGWELCCEWDE-W

magus2_083 MRCLPVFVILLLLIASAPSADARLKTKDDMPLPSSHANIKRTLQMLRNKRCCPGWELCCEWDDGW

marmoreusADZ99330 MRCLPVFVILLLLIASAPSVDARLKTKD-MPLPSSHANIKRTLQIHRNKRCCPGWELCCEWDDWW

magus1_090 MYCLPVFLILLLLISSAPSAPPQPRNKDRVHLVSLLDNQKQILQRDWNGCCAKKAGCCSWGK

imperialisQ9U6Z5 MYCLPVFIILLLLISSAPSTPPQPRNKDRVHLISLLDNHKQILQRDWNSCCGKNPGCCPWGK

magus1_093 MCCIPVFFILLLLIPSAPSILAQPTTKGDVALASSYDDAKRTLQRLSIKYSCCPGIVSCCVIP

imperialisAME17682 MCCIPVFFILLLLIPSAPSILAQPTTKGDVALASSYDDAKRTLQRLSIKYSCCPGIVSCCVIP

magus1_093

imperialisAME17682

magus1_094 -----VFIILLLLVPSTPSVDVQPFTKNDVTLDSLRNVATKPLQRLLNTRCCIKFHPCCHNG

imperialisADZ99324 MYCLPVFIILLLLIPSAPSVDVQPITKNDVILDSLRNVATKPLQRLLNTRCCIKFHPCCHNG

magus2_085 MRCLPVLIILLLLTASAPGVYVLPKTEDDVPLSSVYGNGKSILRGILRNGVCCGYKLCHPC

marmoreusBAO02249 MRCLPVLIILLLLTASAPGVVVLPKTEDDVPLSSVYGNGKSILRGILRNGVCCGYKLCHPC

magus1_089 MHYLPVFVILLLLTASGPSVDAGLKTKDDVPLSSFRDNAKSTLRRLQYKQACCGFKMCVPCG

episcopatusBAS25471 MYCLPVFVILLLLIASAPSVDALLKTKDDVPLSSFRDNAKSTLQRLQDKSTCCGYRMCVPCG

textileP0DPL8 MHCLPIFVILLLLTASGPSVDAQLKTKDDVPLSSFRDHAKSTLRRLQDKQTCCGYRMCVPCG

magus1_102 MRCLPVVVFLLLLLSAAAAPGVGSKTERLPGLTSSGDSDESLPFLNTICCWSGACCGG

imperialisADZ74140 MRCLPVVVFLLLLLSAAAAPGVGSKTERLPGLTSSGDSDESLPFLNTICCWSGACCGG

magus1_102

imperialisADZ74140
